# Supplementary material for: The Emerging Role of NANOG as an Early Cancer Risk Biomarker in Patients with Oral Potentially Malignant Disorders
Source: J Clin Med. 2019 Sep 3;8(9):1376. doi: 10.3390/jcm8091376 (PMC6780631; doi:10.3390/jcm8091376)
Supplement: Supplementary file 1 [file jcm-08-01376-s001.pdf]

**Supplementary Table S1.** Clinical and pathological characteristics of the cohort of 125 patients with OSCC selected for study.

| Variable | Number (%) |
|---|---|
| Age (year) (mean ±SD; median; range) | 58.69 ± 14.34; 57; 28 - 91 |
| Gender | |
| Men | 82 (66) |
| Women | 43 (34) |
| Tobacco use | |
| Smoker | 84 (67) |
| Non-smoker | 41 (33) |
| Alcohol use | |
| Drinker | 69 (55) |
| Non-drinker | 56 (45) |
| Location of oral squamous cell carcinoma | |
| Tongue | 51 (41) |
| Floor of the mouth | 37 (30) |
| Other sites within the oral cavity | 37 (30) |
| Tumor status | |
| pT1 | 27 (22) |
| pT2 | 54 (43) |
| pT3 | 16 (13) |
| pT4 | 28 (22) |
| Nodal status | |
| pN0 | 76 (61) |
| pN1 | 25 (20) |
| pN2 | 24 (19) |
| Clinical stage | |
| Stage I | 20 (16) |
| Stage II | 32 (26) |
| Stage III | 26 (20) |
| Stage IV | 47 (38) |
| G status | |
| G1 | 80 (64) |
| G2 | 41 (33) |
| G3 | 4 (3) |
| Second primary carcinoma | |
| No | 106 (85) |
| Yes | 19 (15) |
| Local recurrence | |
| No | 71 (57) |
| Yes | 54 (43) |
| Clinical status at the end of the follow-up | |
| Alive and without recurrence | 53 (42) |
| Dead of index cancer | 53 (42) |
| Lost or died of other causes (censored) | 19 (16) |

**Supplementary Table S2.** Cross-tab to evaluate the correlation between NANOG and PDPN protein expression in OPMDs. Positive PDPN expression was defined as positive staining in one or more dysplastic areas beyond basal cell layer (scored as 2 and 3) as described in [25].

| | | **PDPN Protein Expression** | | |
|---|---|---|---|---|
| | | **Negative** | **Positive** | ***Total*** |
| **Cytoplasmic NANOG Expression** | **Score 0** | 6 (32) | 13 (68) | 19 (100) |
| | **Score 1** | 1 (50) | 1 (50) | 2 (100) |
| | **Score 2** | 5 (100) | 0 (0) | 5 (100) |
| *Total* | | 12 (46) | 14 (54) | 26 (100) |

Figures are number of cases and percentages between brackets. $P = 0.017$, Fisher exact Test.
